# Cloud‐based archived metabolomics data: A resource for in‐source fragmentation/annotation, meta‐analysis and systems biology

**DOI:** 10.1002/ansa.202000042

**Published:** 2020-06-13

**Authors:** Amelia Palermo, Tao Huan, Duane Rinehart, Markus M. Rinschen, Shuzhao Li, Valerie B. O'Donnell, Eoin Fahy, Jingchuan Xue, Shankar Subramaniam, H. Paul Benton, Gary Siuzdak

**Affiliations:** ^1^ Scripps Center for Metabolomics The Scripps Research Institute La Jolla California USA; ^2^ Department of Chemistry University of British Columbia Vancouver British Columbia Canada; ^3^ Department of Bioengineering University of California San Diego La Jolla California USA; ^4^ Department of Chemistry Molecular and Computational Biology The Scripps Research Institute La Jolla California USA; ^5^ The Jackson Laboratory for Genomic Medicine Farmington Connecticut USA; ^6^ Systems Immunity Research Institute Cardiff University Cardiff UK

**Keywords:** archived data, meta‐analysis, metabolic pathways, metabolomics, proteomics, systems biology, transcriptomics

## Abstract

Archived metabolomics data represent a broad resource for the scientific community. However, the absence of tools for the meta‐analysis of heterogeneous data types makes it challenging to perform direct comparisons in a single and cohesive workflow. Here, we present a framework for the meta‐analysis of metabolic pathways and interpretation with proteomic and transcriptomic data. This framework facilitates the comparison of heterogeneous types of metabolomics data from online repositories (eg, XCMS Online, Metabolomics Workbench, GNPS, and MetaboLights) representing tens of thousands of studies, as well as locally acquired data. As a proof of concept, we apply the workflow for the meta‐analysis of (a) independent colon cancer studies, further interpreted with proteomics and transcriptomics data, (b) multimodal data from Alzheimer's disease and mild cognitive impairment studies, demonstrating its high‐throughput capability for the systems level interpretation of metabolic pathways. Moreover, the platform has been modified for improved knowledge dissemination through a collaboration with Metabolomics Workbench and LIPID MAPS. We envision that this meta‐analysis tool combined with our in‐source fragmentation/annotation (ISA) technology will help overcome the primary bottleneck in analyzing diverse datasets and facilitate the full exploitation of archival metabolomics data for addressing a broad array of questions in metabolism research and systems biology.

## 1 INTRODUCTION

Metabolites are the prime drivers of biological activity as they regulate enzyme reactions,^1^ protein activation, and gene/protein expression.^2^ Ultimately, metabolites provide an accessible functional readout for the activity of the system and in themselves modulate the phenotype.^3^ In line with this, the meta‐analysis of untargeted high‐resolution mass spectrometry (MS) metabolomic data obtained from distinct studies can be used to obtain a better understanding of the altered metabolic processes and active endogenous metabolites affecting the system over a broad population of samples. This type of analysis requires the generation and/or recollection of multiple metabolomic data sets across several independent studies, to provide a more comprehensive picture than an individual study. In some cases, the data sets required for the meta‐analysis have already been generated and made available on public databases. In this regard, several data storage infrastructures have been recently developed to address the raising call for metabolomics data sharing and currently encompass more than 1000 untargeted high‐resolution data sets. Emerging open‐access ecosystems include MetaboLights,^4^ MetabolomicsWorkbench,^5^ Metabolonote,^6^ Global Natural Products Social Molecular Networking (GNPS),^7^ and metabolomic data aggregation services, such as metabolomeXchange^8^ (http://www.metabolomexchange.org/site/) and Omics Discovery Index (http://www.omicsDI.org).^9^ In addition, the LIPID MAPS service provides a link into MetabolomicsWorkbench to support the direct deposition of lipidomics data (www.lipidmaps.org).^10^, ^11^

These publicly available data sets can reduce the workload for data re‐collection as well as foster transparency and collaboration between researchers. However, owing to the absence of tools for their cohesive meta‐analysis and to the heterogeneity of the stored data, that are often obtained by different types of MS‐based metabolome profiling workflows, each study remains only partially utilized for comparative analyses.

Currently, the meta‐analysis of metabolomic pathways is carried out by comparing and analyzing the results reported in published papers (*eg*, fold change comparison, absolute concentrations from targeted studies), thus ignoring the total content of information on metabolites contained in the raw profiling data. Moreover, the interpretation of the meta‐analysis findings in the context of proteomic and transcriptomic dysregulations remains a manual task as no systems level data interpretation tool currently provides this functionality. For example, depending on data type, there are many tools for integration of multi‐omics data available including correlation analysis, multivariate comparison, regression/machine learning for sample classification.

Here, we report MetaXCMS framework, to enable the meta‐analysis of heterogeneous types of archived untargeted, high‐resolution MS data across metabolomics, proteomics, transcriptomics, and genomics. The XCMS Online metabolomics platform^12^, ^13^ is an environment for the direct re‐analysis, in‐source fragmentation/annotation^47^, ^48^ and comparison of data from transcriptomics and metabolomics repositories and/or acquired locally, to gather insights into the dysregulated active metabolites and pathways over independent studies and populations of subjects/samples. We deploy this workflow by integrating and interpreting at systems level archival data sets from two independent colon cancer studies obtained from the XCMS Online Public repository.^14^ In addition, we tested this framework in the meta‐analysis of archival multimodal metabolomics data acquired from plasma samples from patients with Alzheimer's disease, mild cognitive impairment, and cognitive normal patients, from the MetabolomicsWorkbench.^15^

## 2 RESULTS

### 2.1 Workflow for the meta‐analysis of archival metabolomic data and systems level integration

**FIGURE 1:**
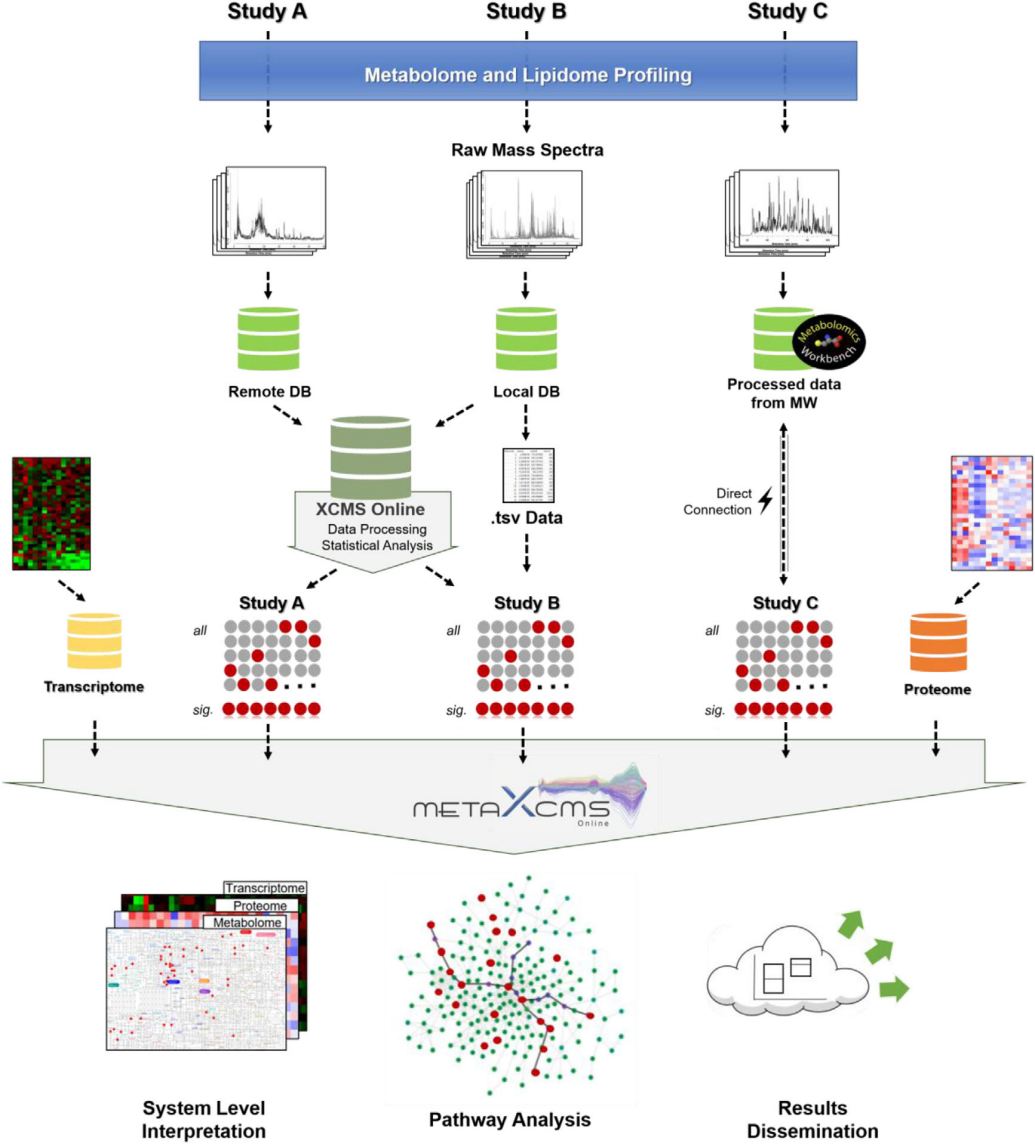
Workflow for the meta‐analysis of heterogenous metabolomics data combined with our in‐source fragmentation/annotation (ISA) technology^47^, ^48^ in XCMS Online: metabolomics data sets from public repositories are uploaded in the XCMS Online database and processed for metabolic features detection and statistical analysis. The jobs are then used as input for meta‐analysis and integration with proteomics and transcriptomics data in Meta XCMS framework. Results can be shared in the XCMS Online cloud for knowledge dissemination

We developed Meta XCMS framework for the meta‐analysis and interpretation of archival metabolomics data by developing and combining different bioinformatic modules to be facilitated with the XCMS Online platform (Figure 1).

In the meta‐analysis workflow, the raw MS data sets from individual studies can be uploaded in XCMS Online to perform data processing and analysis, including peak detection, retention time alignment, putative annotation, and statistical significance testing, to a final list of detected and dysregulated metabolic features.^13^ At this level, the user can set the processing and statistical parameters depending on the analytical platform employed for metabolome profiling and on the statistical test needed for that study. The processed jobs can then be selected and downloaded to be inputted into the Meta XCMS framework for further analysis. Multiple metabolomic analysis (analytical modalities) for a given study can be combined together for comprehensive coverage, for example, on lipid and central carbon metabolism *(eg*, combining data obtained by reversed phase chromatography coupled with positive electrospray ionization (ESI)–MS, and hydrophilic interaction liquid chromatography in negative ESI–MS, etc).^16^, ^17^ Moreover, metabolomics data sets obtained from high‐resolution untargeted studies archived in the Metabolomics Workbench can be directly uploaded for meta‐analysis, and the user interface also supports the upload of metabolomics data sets in text/tsv format, obtained through alternative data preprocessing workflows.

The Meta XCMS framework code is based on mummichog version 2.0.7^17^, ^18^ and leverages itself on this open source platform. Briefly, the mummichog algorithm performs a Fisher's exact test on the number of metabolites jointly dysregulated in the studies as opposed to the total metabolites in the pathway, to predict active pathways directly from putative metabolic features. To allow for a multi‐file input, the code and algorithm were adjusted. Using either data from the Metabolomics Workbench or XCMS Online, data are read into the system via convenient tsv/csv peak list file formats. Data read into the system are first parsed, each feature is tagged to its input file to allow for tracing throughout the system. Several possible adducts combinations are calculated onto the feature masses. These features are queried against the pathway database, each file is processed separately and ranked on their corresponding *P*‐value score to statistically eliminate false positives. Once a list of possible hits is obtained, features that match the neutral mass in the pathway, they are merged between the different files. This is done such that any compound that is seen in more than one file is merged together and the best *P*‐value score is taken. This method allows for the expanded coverage and keeps the statistical validation. Next, the regular *mummichog* process continues with the statistical validation of the matched pathways. Finally, the output is processed to simplify future analyses.

For each individual study included in the meta‐analysis, the user can set a specific significance threshold (*P*‐value), *m/z* tolerance for metabolite putative annotation, and filter the metabolic feature list according to a specified intensity threshold. We recommend to carefully choose these parameters considering the size of each individual study and the type of metabolomics platform used.

Notably, lipids comprise around a third of all metabolites, but they require distinct processing approaches for accurate annotation and pathway prediction. In particular, removing spurious MS signals is critical to improve statistical power, especially for lipids where multiple forms of the same molecule can be detected/exist. LipidFinder^19^ has been recently developed at LIPID MAPS^11^ to alleviate these artifacts. Here, we suggest using LipidFinder post processing of XCMS outputs. This helps to further the broaden the output of lists of putative structures and their categories for more accurate meta‐analysis of lipidomics data.

As part of the Meta XCMS framework, we suggest performing multi‐omic data integration by superimposing user‐uploaded transcriptomic and proteomic data sets onto the dysregulated pathways. Using a list of dysregulated genes (as gene symbols or loci) and proteins (as UniProt accession IDs or gene symbols) obtained from studies targeting a given biological question users can generate improved confidence on the pathway hits. The integration with proteomics and transcriptomics results offers the possibility to gauge a systems level mechanistic understanding of pathways dysregulation and metabolite activity in the investigated biological system.^20^

The downloaded “metabolite results” table reports the list of all the overlapping dysregulated metabolites detected in the studies included in the meta‐analysis and used for pathway prediction.

The “pathway results” table showcases the output for the metabolic pathways jointly dysregulated in the studies. For each metabolic pathway, it reports the number of overlapping dysregulated metabolites detected in different studies with respect to the total number of metabolites in the pathway. By clicking on the number of “shared metabolites” the complete list of metabolic features is shown. Entries can be further filtered based on the adduct type, study group or pairwise job group. Moreover, this table reports the overlapping dysregulated genes and proteins from the uploaded proteomics and transcriptomics data for each metabolic pathway, thus providing a rapid glance on the biological process from a system‐wide perspective.

### 2.2 Expanding the capability of XCMS Online for the meta‐analysis of archived data

**FIGURE 2:**
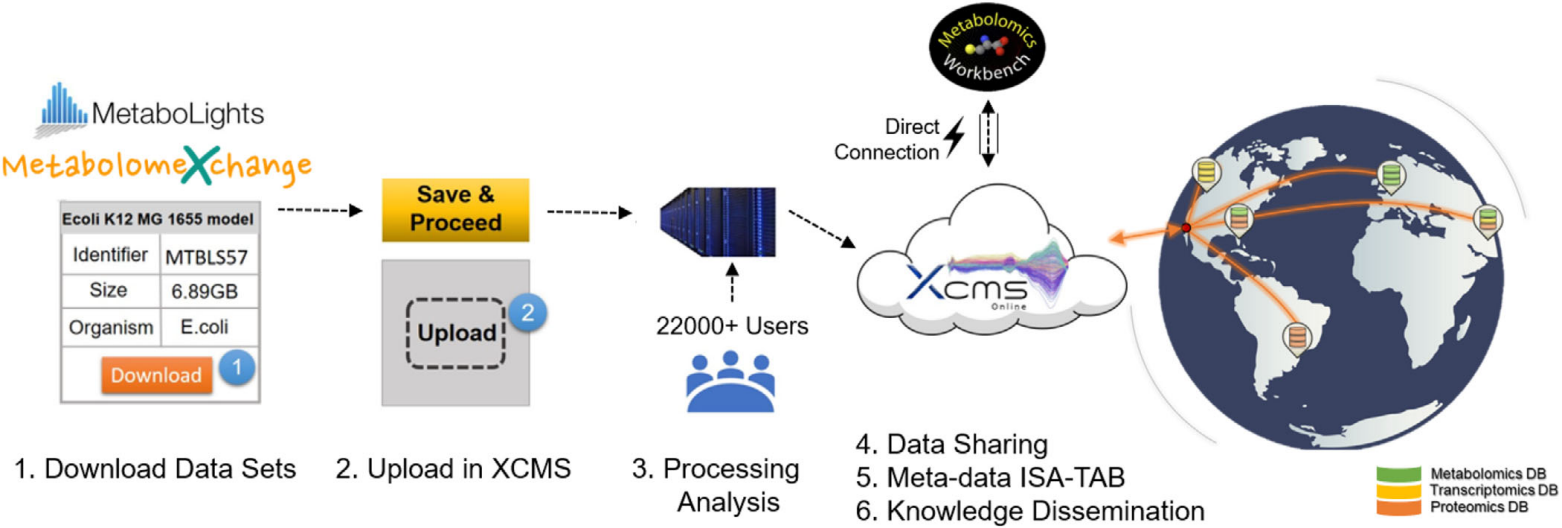
Developments of the meta XCMS framework to enhance archival metabolomics data processing, archiving and sharing for meta‐analysis and systems level interpretation

To allow the meta‐analysis of metabolomics data sets obtained from disparate sources, we enabled easy parsing file options of tsv/csv in Meta XCMS framework (Figures 1 and 2). First, the user can select studies processed in the XCMS Online private space or in the XCMS Online Public. These data are then easily downloaded to be parsed into the Meta‐XCMS framework. Alternatively, the Metabolomics Workbench data can also be used. On many of the studies there are already existing outputs of identified metabolites or feature lists. In these instances where a metabolite is already identified it will be read into the system and used as a confirmed metabolite of the pathway analysis.

This strategy is aimed at fostering data dissemination and at actively promoting the full exploitation of archived metabolomics data through stimulating further meta‐analysis for results validation or for generating novel hypothesis.

### 2.3 Analysis of archived metabolomics data

We tested the workflow in the systems level meta‐analysis of two independent colon cancer tissue metabolomics data sets by leveraging archival data from the XCMS Online Public repository, and for the meta‐analysis of Alzheimer's disease and mild cognitive impairment studies in plasma, including heterogeneous profiling data from the Metabolomics Workbench.

#### 2.3.1 Colon cancer

**FIGURE 3:**
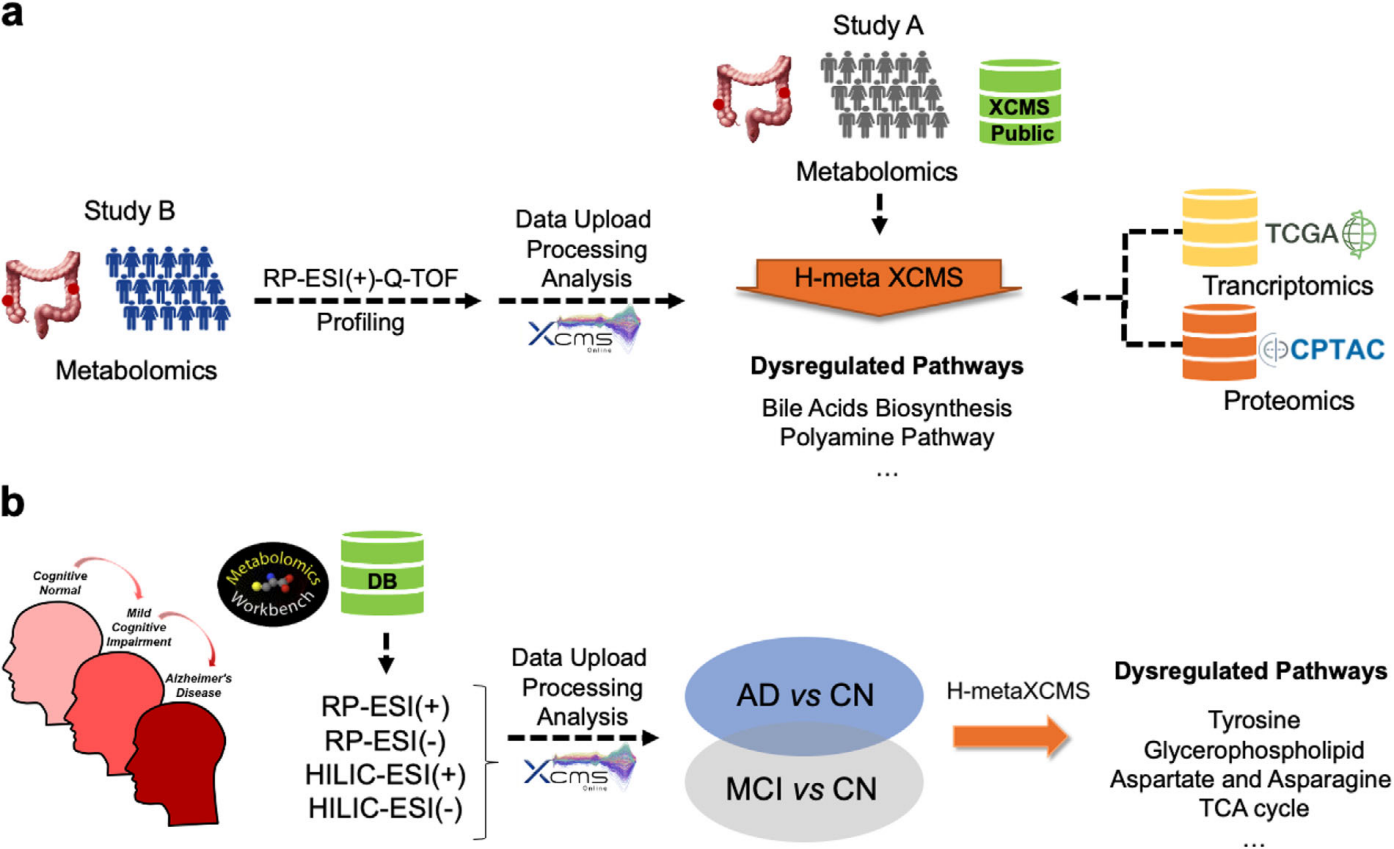
a, Meta‐analysis of colon cancer metabolomics studies, and systems level interpretation with proteomics and transcriptomics data; b, cohesive re‐analysis of heterogeneous archival metabolomics data from AD and MCI, compared with CN

Several previous studies have pinpointed the multifaced metabolic reprogramming underlining colon cancer.^14^, ^24^, ^25^ Here, we performed the meta‐analysis of archived untargeted metabolomics data from a study investigating the role of bacterial biofilms in colon cancer^14^ (Study A) and a second colon cancer study, recently performed in our laboratory (Study B) (Figure 3a). Study A involved 30 subjects diagnosed with colon cancer from stage 3 to 4 (18 females and 12 males, 61‐88 years old) and was available in the XCMS Online Public repository,^14^ while Study B involved 19 subjects diagnosed with colon cancer (13 females and 6 males, 62‐92 years old). More details on study design and samples collection are available in Table S1. Both studies used similar platforms for metabolome profiling (reversed phase chromatography coupled with ESI(+)‐quadrupole time‐of‐flight (Q‐TOF) MS) and sample preparation protocols, therefore we expected comparable metabolome coverage and overlapping dysregulations (excluding inter‐population heterogeneity).

**FIGURE 4:**
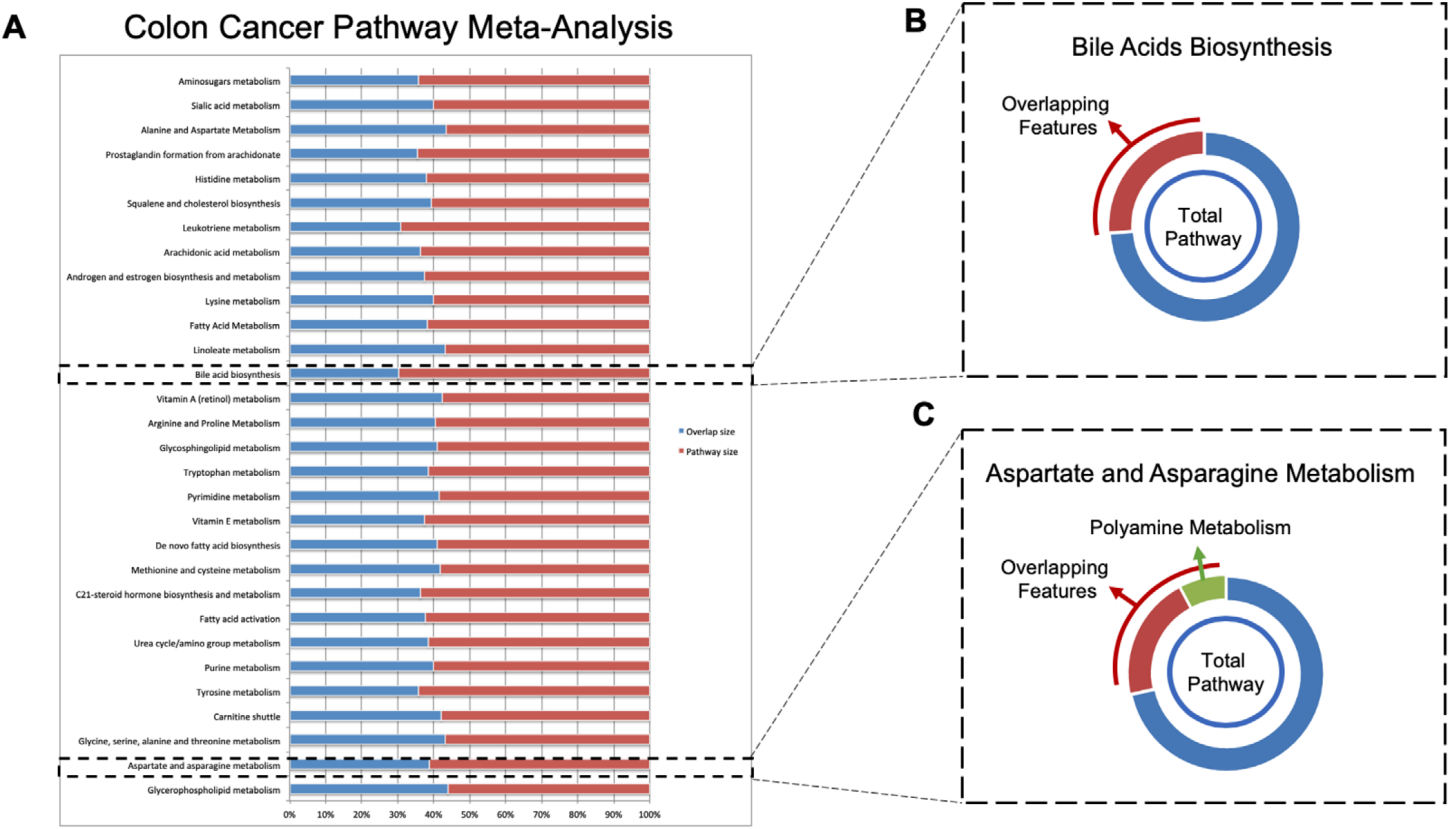
Meta‐analysis across independent colon‐cancer studies predicts 30 dysregulated metabolic pathways (a), including bile acids biosynthesis (b) and polyamine metabolism (c)

First, raw data from study B were uploaded in XCMS Online and processed as a paired job. This job and the archival job (Study A) were then selected as input for meta‐analysis in meta XCMS framework (Figure 3a). The results unveiled the presence of 30 metabolic pathways with at least 10 dysregulated putative metabolic features across study A and B (Table S2; Figure 4a), among these are glycerophospholipids metabolism, aspartate and asparagine metabolism, glycine, serine and alanine metabolism, carnitine shuttle, tyrosine metabolism, steroidal hormones, and bile acids. The dysregulation of the glycerophospholipid metabolism has been previously confirmed correlating with altered viability, proliferation, and colorectal cancer development.^26^ The meta‐analysis also highlighted the dysregulation of the aspartate and asparagine pathway that includes spermine/spermidine biosynthesis and degradation (polyamine pathway) where N1‐acetylspermidine, N1‐acetylspermine, spermidine, and N1,N12‐diacetylsperimine, spermidine dialdehyde, spermic acid were found jointly upregulated, a finding consistent with previous work (Figure 4c).^14^, ^30^ Of note, in the bile acids biosynthesis pathway taurine and taurochenodeoxycholate were upregulated (Figure 4b). Increased levels of conjugated bile acids have been previously reported to highly associate with colon cancer.^27^ In particular, taurochenodeoxycholate can be hydrolyzed releasing taurine, a sulfur amino acid further transformed by the gut microbiota to form compounds with genotoxic activity (eg, H_2_S), and colon tumor promoters (deoxycholic acid).^28^, ^29^

To Interpret this evidence in light of the variations occurring at proteomic and transcriptomic level, colon cancer data sets obtained from The Cancer Genome Atlas^24^ and The CPTAC Proteomics Data Portal^32^ were uploaded and processed in meta XCMS framework. Approximately 90% of the upregulated metabolic pathways were further supported by dysregulated proteins and gene transcripts (Table S3). For instance, both polyamines and bile acids pathway dysregulations were confirmed (Figure 3a).

#### 2.3.2 Alzheimer's disease

Alzheimer's disease (AD) is a progressive neurodegenerative disorder of unknown etiology.^33^ AD and dementia patients are usually subject to a long pre‐AD period known as mild cognitive impairment (MCI). Here, we used public available metabolomics data obtained from previous longitudinal studies performed at the Mayo Clinic Study of Aging (MCSA) and Mayo Clinic Alzheimer Disease Research Center (ADRC).^15^ Plasma samples were from AD, MCI, and cognitive normal (CN) subjects (15 individuals/group). The metabolomics data sets and meta‐data were publicly available in the Metabolomics Workbench and formerly generated by LC‐Q‐TOF MS in four analytical modalities (hydrophilic interaction liquid chromatography [HILIC] and reversed phase liquid chromatography [RP], in both positive and negative ESI modes) for comprehensive metabolome coverage. We downloaded the raw data sets from the Metabolomics Workbench repository and uploaded them in the XCMS Online for processing and statistical analysis to extract significant metabolic variations in AD versus CN and MCI versus CN. The resulting XCMS Online jobs were then used as input for the Meta XCMS framework to detect shared metabolic changes at different disease stages.

**FIGURE 5:**
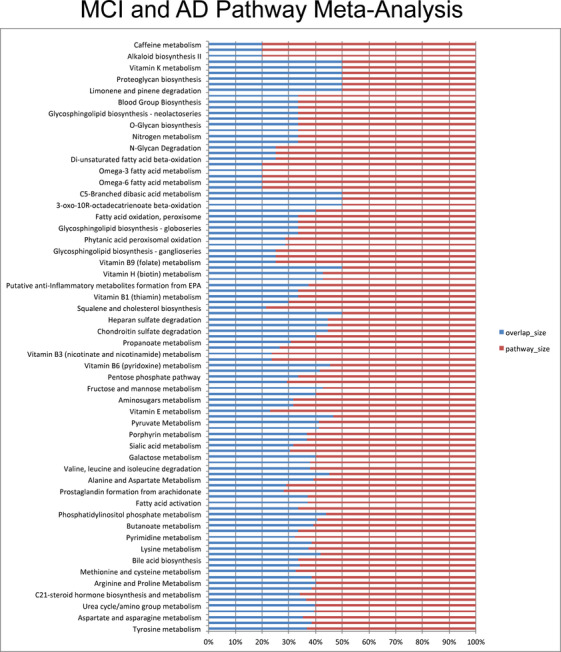
Meta‐analysis across AD versus CN and MCI versus CN studies in plasma predicts 101 dysregulated metabolic pathways

Meta XCMS framework predicted 24 dysregulated metabolic pathways with at least 10 metabolic features dysregulated in the AD versus CN and the MCI versus CN groups, over a total of 101 paths (Table S4). We manually compared the output with the dysregulated pathways reported in the original publication.^15^ Our development predicted the dysregulation of tyrosine, glycerophospholipid, aspartate and asparagine, glycine, serine and alanine metabolism, urea cycle, tryptophan, and purine metabolism, together with the other pathways reported in Table S4. The original work reported 50 total dysregulated pathways, of which nine were consistently predicted across AD versus CN and MCI versus CN (Figure 5). Our approach predicted a total of 101 dysregulated pathways, of which 24 pathways were reported in the original publication, demonstrating the efficiency of the workflow in identifying jointly dysregulated metabolic pathways from heterogeneous archived metabolomic data.

## 3 DISCUSSION

Archived metabolomics data are a rich source of information for second‐order analysis by the scientific community. However, the heterogeneity of the data and the lack of tools for their cohesive re‐analysis and interpretation hinders their full utilization. To address this, we developed a framework for archival data re‐processing, analysis, integration, and interpretation at systems level. The workflow moves from the metabolomics tools available in the XCMS Online, further combining them with a bioinformatic development specifically designed for the meta‐analysis of heterogeneous metabolomics data.

A key aspect of the workflow is the use of a pathway‐centric approach to the meta‐analysis, which allows the direct prediction of the dysregulated metabolic pathways from putative metabolic features jointly detected in the archived data/studies. This is performed through the embedment of a recently developed tool for metabolic pathway prediction from putative annotations of metabolic features extracted from different types of metabolomics data sets.^17^, ^18^ This tool allows for higher confidence in the putative pathway enrichment results by estimating the probability of a pathway being dysregulated on the basis of the total number of dysregulated metabolites detected. It is also worth noting that, when attempting to re‐analyze archival metabolomics data, the physical samples are not directly accessible and often no longer available. This makes it unfeasible to perform further MS fragmentation experiments and metabolite identity confirmation. In this scenario, performing direct pathway prediction analysis represents a practical strategy to bypass this limitation and directly formulate biological hypothesis from the archived data, to be later tested through independent targeted studies or biochemical assays.

The use of a pathway‐centric approach also streamlines the interpretation of the metabolomic data by superimposing the dysregulated proteins and transcripts to each metabolic pathway. This provides a rapid glance on the system in the light of other omics regulatory levels, introducing the possibility for the orthogonal confirmation of the insights extracted from the archived metabolomics data.

Despite recent efforts aimed at standardizing metabolome profiling and reporting,^21^, ^35^, ^36^ a widely adopted consensus on untargeted MS‐based workflows in the perspective of meta‐analysis is still missing.^37^, ^38^ The metabolic profiles are indeed usually acquired by a variety of different analytical solutions,^16^, ^39^, ^40^, ^41^ thus introducing heterogeneity in the type of available data. For example, Metabolomics Workbench^5^ currently stores >190 untargeted high‐resolution MS studies, for a total of ∼400 different analyses (ie, different analytical modalities including ESI positive and negative acquisition modes), while Metabolights^4^ stores ∼350 among GC‐ and LC‐MS based studies. This heterogeneity complicates the development of bioinformatics solutions for the automated meta‐analysis, as each different metabolomics platform calls for specific data processing and metabolite annotation pipelines. To circumvent this limitation, we designed the workflow in a modular fashion: each study can be processed as pairwise XCMS Online job using different processing and statistical settings and the resulting jobs can be used as input for further meta‐analysis. This allows the use of raw data acquired by different metabolomics platforms and modalities. For example, the user can upload both lipidomics and metabolomics data obtained by different chromatographic or ionization modes for comprehensive metabolome coverage and improved pathway prediction.^17^ This, together with the ability to perform multi‐omics data integration, represents a fundamental advantage over our previous development for the meta‐analysis of metabolomics data.^42^, ^43^

Besides raw data sets, the workflow also supports the direct comparison of untargeted studies already processed and available in the XCMS Online Public cloud and in the Metabolomics Workbench. Of note, several data sets currently archived in public databases are not compliant with the ISA guidelines for meta‐data reporting, unearthing the need of harmonized and more pragmatic guidelines for metabolomics data sharing in public repositories.^37^

The meta‐analysis of metabolomics data sets and their interpretation at systems level has the potential of streamlining different types of study comparisons. For example, a meta‐analytical approach can be used for (a) providing further validation of metabolite dysregulations in the context of independent set of samples (*eg*, in biomarker studies); (b) stimulating the generation of biological hypothesis from the re‐analysis of archived untargeted studies; (c) streamlining the exclusion of experimental artifacts to reduce the list of dysregulated metabolites before performing time consuming structural elucidation^42^; (d) excluding metabolic dysregulations due to physiologic heterogeneity in different populations of subjects, therefore taking a step towards the identification of therapeutic targets and biomarkers of broad applicability. In particular, in biomarker discovery the automatic integration of multiple archival metabolomics studies can be a cost‐effective strategy to minimize the interstudy bias introduced by genetic and environmental factors.^44^, ^45^, ^46^ This strategy is not limited to archived data, since the difficulty in cross‐laboratory comparison has impeded the biomedical applications of metabolomics. In an emergency situation like the current COVID‐19 pandemic, the pathway‐centric meta‐analysis can be important for identifying scientific consensus in a timely manner.

As proof of concept, we demonstrated the utility of the workflow in two meta‐analytical studies. First, we leveraged archival data sets from a previous study available in the XCMS Online Public,^14^ for the autonomous comparison with a colon cancer study recently performed in our laboratory. The workflow allowed a rapid glance on metabolic pathways jointly dysregulated and validation at systems level (*eg*, the bile acid and the polyamine pathways). In the second example, we applied the workflow for the re‐analysis of archival biomarker studies obtained from the Metabolomics Workbench database. The workflow permits the streamlined and autonomous prediction of metabolic pathways changed in both AD and MCI patients in plasma (pre‐AD) in agreement and beyond the results previously obtained by manual meta‐analysis.^15^

In summary, there are many challenges in the analysis of diverse datasets including variability in experimental designs as well as information types that are largely platform dependent. However, by combining a fully automated workflow including in‐source fragmentation/annotation^47^, ^48^ with an enhanced strategy for data storage and direct connection to the Metabolomics Workbench data repository, the described approach can provide a solution for meta‐analysis, with the ultimate goal of maximizing the usage and dissemination of information‐rich archival metabolomics data. With the growing number of metabolomics, proteomics and genomics data generated to cover a wide range of biological questions, this workflow paves the way to unlock biological insights in the era of “big data” and “open science.”

## 4 MATERIALS AND METHODS

### 4.1 Meta‐analysis framework

The system has been built on a local based Flask system with code based on mummichog version 2.0.7 running on python 2.7.^18^ Several files were altered to allow for a multi‐file input and hosting on a web frontend. Metabolomics Workbench data is processed using either csv/tsv formats directly into the software or via XCMS processing to csv/tsv files. Once the data are read each feature is processed against all possible adducts for search masses. Using the mummichog algorithm, the search masses are searched against the pathway database, each file is processed separately and ranked on their corresponding *P*‐value score. Once a list of possible hits is obtained, they are merged between the different files such that any compound that is seen in more than one file is merged together and the best *P*‐value score is taken. Now, the regular mummichog process continues with the statistical validation of the altered pathways. Finally, the output is processed to make further analysis simpler and result are downloaded by the user. Framework code has been made available via github at https://github.com/hpbenton/archive-mummi

### 4.2 Metabolomics profiling data

The colon cancer study A was available in the XCMS Online Public Space as a processed job. The raw MS data from high‐resolution metabolome profiling for the colon cancer study B were archived in our laboratory, and previously obtained as part of a pilot colon cancer study (Study B). Both profiling studies were performed in RP‐ESI(+)‐Q‐TOF profiling. More details on study A and B experimental setups can be found in previous published work.^14^, ^17^ Metabolome profiles for AD, MCI, and CN plasma samples were downloaded from the Metabolomics Workbench repository,^5^ uploaded and re‐processed in the XCMS Online. These studies were performed at the Mayo Clinic Study of Aging ((MCSA) and Mayo Clinic Alzheimer Disease Research Center (ADRC) and more details on study design and experimental procedures are available in previous published work.^15^

### 4.3 Data processing and re‐analysis

Raw archival data sets were uploaded as .mzXML files in the XCMS Online and processed as pairwise jobs. Before processing, the profiling data were manually examined for assessing the quality and the parameters for further processing and analysis. Colon cancer study A was already processed and available in the XCMS Online Public. The XCMS jobs were used for further meta‐analysis in the Meta XCMS framework. *P*‐value, intensity, and ppm error settings are reported in Supporting Information Text S1.

### 4.4 Proteomics and transcriptomics data

Transcriptomics data were obtained from The Cancer Genome Atlas (TCGA)^31^ in the frame of a previous colon cancer study involving 22 subjects (22 colon cancer tissue samples vs 22 paired normal tissues). Dysregulated genes were selected based on a *P*‐value cut off of 0.01 and fold change > 4. A total of 7138 dysregulated transcripts were included in the final data set for upload in the XCMS Online as gene symbols. Proteomics data were obtained by the Clinical Proteomic Tumor Analysis Consortium (CPTAC), involving 90 patients affected by colon cancer (90 colon cancer tissue samples and 90 paired normal tissues). Dysregulated proteins were filtrated by *P*‐value < .01 and fold change > 2, obtaining a total of 2545 dysregulated proteins uploaded in the XCMS Online as UniProt accession IDs for multiomic analysis.

## CONFLICT OF INTEREST

The authors declare no conflict of interest.

## DATA AVAILABILITY

As referred to in the text, the data is available through multiple sources.

## Supporting information

Supporting Information
