# Supplementary material for: Cloud‐based archived metabolomics data: A resource for in‐source fragmentation/annotation, meta‐analysis and systems biology
Source: Anal Sci Adv. 2020 Jun 13;1(1):70–80. doi: 10.1002/ansa.202000042 (PMC8858440; doi:10.1002/ansa.202000042)
Supplement: Supplementary file 1 — Supporting Information [file ANSA-1-70-s001.docx]

**Supporting Information**

To

**Archived Metabolomics Data in the Cloud:**

**A Resource for Meta Analysis and Systems Biology**

Amelia Palermo^1^, Tao Huan^1,2^, Duane Rinehart^1^, Jingchuan Xue^1^, Markus M. Rinschen^1^, Shuzhao Li^5^, Valerie O. Donnell^6^, Eoin Fahy^3^, Jingchuan Xue^1^, Shankar Subramaniam^3^, H. Paul Benton^1^*, Gary Siuzdak^1,4^*

^1^ Scripps Center for Metabolomics, The Scripps Research Institute, 10550 North Torrey Pines Rd., La Jolla, CA, 92037, USA

^2^ Department of Chemistry, University of British Columbia, 2036 Main Mall, Vancouver, BC, V67 1z1, Canada

^3^ Department of Bioengineering, University of California San Diego, 9500 Gilman Dr, La Jolla, CA, 92093-0412, USA

^4^Department of Chemistry, Molecular and Computational Biology, The Scripps Research Institute, 10550 North Torrey Pines Rd., La Jolla, CA, 92037, USA

^5^Department of Medicine, School of Medicine, Emory University, 615 Michael Street, Atlanta, GA 30322-1047, USA

^6^Systems Immunity Research Institute, Cardiff University, Sir Martin Evans Building, Museum Avenue, Cardiff CF10 3AX, UK

*Author to whom correspondence should be addressed:

Gary Siuzdak

Tel: (858) 784-9415

E-mail: siuzdak@scripps.edu

Internet: <https://masspec.scripps.edu/>

**Index**

Tables

Table S1: colon cancer study A and B meta-data……................…………………………………………………………...…2

Table S2: predicted pathways jointly dysregulated in colon cancer study A and B…………………………....…..4

Table S3. Predicted pathway dysregulation ranked by number of dysregulated genes (top entries)........7

Table S4: predicted pathways jointly dysregulated in the AD and MCI studies…………………………………......8

| **Patient** | **Age** | **Male/Female** | **Localization** |
| --- | --- | --- | --- |
| 1 | 62 | female | colon (left) |
| 2 | 86 | male | colon (right) |
| 3 | 85 | male | colon (right) |
| 4 | 73 | male | colon (right) |
| 5 | 58 | male | colon (right) |
| 6 | 68 | female | colon (left) |
| 7 | 81 | male | colon (right) |
| 8 | 89 | female | colon (left) |
| 9 | 70 | male | colon (right) |
| 10 | 44 | female | colon (left) |
| 11 | 78 | male | colon (right) |
| 12 | 79 | female | colon (left) |
| 13 | 75 | male | colon (right) |
| 14 | 62 | male | colon (right) |
| 15 | 75 | male | colon (right) |
| 16 | 72 | female | colon (left) |
| 17 | 70 | female | colon (left) |
| 18 | 66 | female | colon (left) |
| 19 | 79 | female | colon (left) |
| 20 | 61 | female | colon (left) |
| 21 | 82 | female | colon (left) |
| 22 | 88 | female | colon (left) |
| 23 | 67 | female | colon (left) |
| 24 | 83 | male | colon (right) |
| 25 | 61 | male | colon (right) |
| 26 | 59 | female | colon (left) |
| 27 | 86 | female | colon (left) |
| 28 | 74 | female | colon (left) |
| 29 | 79 | female | colon (left) |
| 30 | 74 | female | colon (left) |
|  | Average | 12 males |  |
|  | 73 | 18 females |  |

**Table S1.** Patient age at time of surgery, gender and tumor localization for colon cancer studies A) and B).

Study A

| **Patient** | **Age** | **Male/Female** | **Localization** |
| --- | --- | --- | --- |
| 1 | 73 | male | cecum (right ) |
| 2 | 75 | male | ascendens (right) |
| 3 | 89 | male | cecum (right) |
| 4 | 66 | female | cecum (right) |
| 5 | 68 | female | ascendens (right) |
| 6 | 81 | male | ceecum (right) |
| 7 | 86 | female | ascendens(right) |
| 8 | 70 | female | cecum (right) |
| 9 | 72 | female | cecum (right) |
| 10 | 77 | female | adenoma ascendens (right) |
| 11 | 92 | female | cecum (right) |
| 12 | 67 | female | cecum (right) |
| 13 | 62 | female | cecum (right) |
| 14 | 89 | female | ascendens (right) |
| 15 | 71 | female | cecum (right) |
| 16 | 81 | female | cecum (right) |
| 17 | 83 | male | cecum (right) |
| 18 | 66 | male | ascendens (right) |
| 19 | 83 | female | ascendens (right) |
|  | Average 76 | 6 males  13 females |  |

Study B

**Table S2.** Predicted pathways jointly dysregulated in colon cancer study A and B**.**

| **Pathway** | **Overlap size** | **Pathway size** |
| --- | --- | --- |
| Glycerophospholipid metabolism | 33 | 42 |
| Aspartate and asparagine metabolism | 28 | 44 |
| Glycine, serine, alanine and threonine metabolism | 25 | 33 |
| Carnitine shuttle | 24 | 33 |
| Tyrosine metabolism | 24 | 43 |
| Purine metabolism | 22 | 33 |
| Urea cycle/amino group metabolism | 22 | 35 |
| Fatty acid activation | 20 | 33 |
| C21-steroid hormone biosynthesis and metabolism | 20 | 35 |
| Methionine and cysteine metabolism | 18 | 25 |
| De novo fatty acid biosynthesis | 18 | 26 |
| Vitamin E metabolism | 18 | 30 |
| Pyrimidine metabolism | 17 | 24 |
| Tryptophan metabolism | 17 | 27 |
| Glycosphingolipid metabolism | 16 | 23 |
| Arginine and Proline Metabolism | 15 | 22 |
| Vitamin A (retinol) metabolism | 14 | 19 |
| Bile acid biosynthesis | 14 | 32 |
| Linoleate metabolism | 13 | 17 |
| Fatty Acid Metabolism | 13 | 21 |
| Lysine metabolism | 12 | 18 |
| Androgen and estrogen biosynthesis and metabolism | 12 | 20 |
| Arachidonic acid metabolism | 12 | 21 |
| Leukotriene metabolism | 12 | 27 |
| Squalene and cholesterol biosynthesis | 11 | 17 |
| Histidine metabolism | 11 | 18 |
| Prostaglandin formation from arachidonate | 11 | 20 |
| Alanine and Aspartate Metabolism | 10 | 13 |
| Sialic acid metabolism | 10 | 15 |
| Aminosugars metabolism | 10 | 18 |
| Beta-Alanine metabolism | 9 | 13 |
| Valine, leucine and isoleucine degradation | 9 | 13 |
| Butanoate metabolism | 9 | 14 |
| Vitamin B3 (nicotinate and nicotinamide) metabolism | 8 | 10 |
| Glycolysis and Gluconeogenesis | 8 | 11 |
| Phosphatidylinositol phosphate metabolism | 8 | 17 |
| Drug metabolism - cytochrome P450 | 8 | 18 |
| Glutamate metabolism | 7 | 9 |
| Glutathione Metabolism | 7 | 10 |
| Propanoate metabolism | 6 | 8 |
| Glycosphingolipid biosynthesis - ganglioseries | 6 | 9 |
| Ascorbate (Vitamin C) and Aldarate Metabolism | 6 | 10 |
| Saturated fatty acids beta-oxidation | 6 | 13 |
| Xenobiotics metabolism | 6 | 20 |
| Nitrogen metabolism | 5 | 5 |
| Hexose phosphorylation | 5 | 6 |
| TCA cycle | 5 | 8 |
| Porphyrin metabolism | 5 | 8 |
| Galactose metabolism | 5 | 8 |
| Selenoamino acid metabolism | 5 | 9 |
| Vitamin D3 (cholecalciferol) metabolism | 5 | 9 |
| Omega-3 fatty acid metabolism | 5 | 10 |
| Vitamin B9 (folate) metabolism | 5 | 11 |
| Chondroitin sulfate degradation | 4 | 4 |
| Vitamin B2 (riboflavin) metabolism | 4 | 4 |
| Heparan sulfate degradation | 4 | 5 |
| Pentose phosphate pathway | 4 | 5 |
| Glycosphingolipid biosynthesis - globoseries | 4 | 5 |
| Dimethyl-branched-chain fatty acid mitochondrial beta-oxidation | 4 | 6 |
| Pyruvate Metabolism | 4 | 6 |
| Ubiquinone Biosynthesis | 4 | 8 |
| Omega-6 fatty acid metabolism | 4 | 10 |
| Phytanic acid peroxisomal oxidation | 4 | 10 |
| Carbon fixation | 3 | 3 |
| Limonene and pinene degradation | 3 | 4 |
| Pentose and Glucuronate Interconversions | 3 | 4 |
| Prostaglandin formation from dihomo gama-linoleic acid | 3 | 4 |
| Vitamin B5 - CoA biosynthesis from pantothenate | 3 | 7 |
| N-Glycan biosynthesis | 3 | 7 |
| Putative anti-Inflammatory metabolites formation from EPA | 3 | 7 |
| Di-unsaturated fatty acid beta-oxidation | 3 | 9 |
| Vitamin B12 (cyanocobalamin) metabolism | 2 | 2 |
| Vitamin H (biotin) metabolism | 2 | 2 |
| Hyaluronan Metabolism | 2 | 2 |
| Lipoate metabolism | 2 | 2 |
| Keratan sulfate biosynthesis | 2 | 3 |
| Vitamin K metabolism | 2 | 3 |
| N-Glycan Degradation | 2 | 3 |
| Keratan sulfate degradation | 2 | 3 |
| Biopterin metabolism | 2 | 3 |
| Glyoxylate and Dicarboxylate Metabolism | 2 | 3 |
| Starch and Sucrose Metabolism | 2 | 5 |
| CoA Catabolism | 2 | 5 |
| Mono-unsaturated fatty acid beta-oxidation | 2 | 6 |
| Drug metabolism - other enzymes | 2 | 6 |
| Vitamin B1 (thiamin) metabolism | 2 | 6 |
| Fructose and mannose metabolism | 2 | 6 |
| Fatty acid oxidation | 2 | 11 |
| Vitamin D | 1 | 1 |
| Proteoglycan biosynthesis | 1 | 1 |
| O-Glycan biosynthesis | 1 | 2 |
| Glycosphingolipid biosynthesis - neolactoseries | 1 | 2 |
| Polyunsaturated fatty acid biosynthesis | 1 | 2 |
| Nucleotide Sugar Metabolism | 1 | 2 |
| Blood Group Biosynthesis | 1 | 2 |
| Glycosphingolipid biosynthesis - lactoseries | 1 | 2 |
| R Group Synthesis | 1 | 2 |
| Dynorphin metabolism | 1 | 3 |
| Benzoate degradation via CoA ligation | 1 | 3 |
| C5-Branched dibasic acid metabolism | 1 | 3 |
| Alkaloid biosynthesis II | 1 | 4 |
| 3-oxo-10R-octadecatrienoate beta-oxidation | 1 | 4 |
| Caffeine metabolism | 1 | 4 |

**Table S3.** Predicted pathway dysregulation ranked by number of dysregulated genes (top entries). ^1^

| **Pathway** | **Overlapping genes** | **Total genes** | **Overlapping proteins** | **Total proteins** |
| --- | --- | --- | --- | --- |
| tRNA charging | 23 | 39 | 18 | 39 |
| glutathione-mediated detoxification | 17 | 24 | 7 | 28 |
| triacylglycerol biosynthesis | 16 | 27 | 5 | 27 |
| 3-phosphoinositide biosynthesis | 14 | 28 | 2 | 29 |
| fatty acid β-oxidation | 13 | 17 | 10 | 24 |
| adenosine ribonucleotides de novo biosynthesis | 13 | 27 | 14 | 27 |
| D-myo-inositol-5-phosphate metabolism | 13 | 20 | 4 | 20 |
| bile acid biosynthesis, neutral pathway | 10 | 14 | 4 | 13 |
| triacylglycerol degradation | 10 | 15 | 1 | 16 |
| retinol biosynthesis | 10 | 18 | 5 | 18 |
| TCA cycle | 10 | 17 | 5 | 12 |
| glycolysis | 10 | 24 | 7 | 24 |
| CDP-diacylglycerol biosynthesis | 10 | 22 | 4 | 22 |
| stearate biosynthesis | 9 | 12 | 8 | 29 |
| γ-linolenate biosynthesis | 9 | 14 | 5 | 14 |
| valine degradation | 9 | 13 | 2 | 10 |
| putrescine degradation III | 9 | 10 | 4 | 10 |
| serotonin degradation | 8 | 9 | 6 | 9 |
| gluconeogenesis | 8 | 24 | 5 | 24 |

1. Huan T, Palermo A, Ivanisevic J, et al. Autonomous Multimodal Metabolomics Data Integration for Comprehensive Pathway Analysis and Systems Biology. *Anal Chem*. 2018;90(14):8396-8403. doi:10.1021/acs.analchem.8b00875

**Table S4.** Predicted pathways jointly dysregulated in the AD and MCI studies.

| **pathway** | **Overlap size** | **Pathway size** |
| --- | --- | --- |
| Tyrosine metabolism | 32 | 55 |
| Glycerophospholipid metabolism | 27 | 43 |
| Aspartate and asparagine metabolism | 24 | 44 |
| Glycine, serine, alanine and threonine metabolism | 24 | 37 |
| Urea cycle/amino group metabolism | 23 | 35 |
| Tryptophan metabolism | 20 | 35 |
| C21-steroid hormone biosynthesis and metabolism | 17 | 33 |
| Purine metabolism | 17 | 27 |
| Arginine and Proline Metabolism | 16 | 24 |
| Carnitine shuttle | 15 | 24 |
| Methionine and cysteine metabolism | 14 | 29 |
| Xenobiotics metabolism | 14 | 27 |
| Bile acid biosynthesis | 13 | 26 |
| Linoleate metabolism | 13 | 18 |
| Lysine metabolism | 12 | 20 |
| Ascorbate (Vitamin C) and Aldarate Metabolism | 12 | 19 |
| Pyrimidine metabolism | 11 | 23 |
| Arachidonic acid metabolism | 11 | 22 |
| Butanoate metabolism | 11 | 17 |
| De novo fatty acid biosynthesis | 11 | 16 |
| Phosphatidylinositol phosphate metabolism | 11 | 14 |
| Drug metabolism - cytochrome P450 | 10 | 20 |
| Fatty acid activation | 10 | 17 |
| Glycosphingolipid metabolism | 10 | 17 |
| Prostaglandin formation from arachidonate | 9 | 23 |
| Androgen and estrogen biosynthesis and metabolism | 9 | 22 |
| Alanine and Aspartate Metabolism | 9 | 14 |
| TCA cycle | 9 | 11 |
| Valine, leucine and isoleucine degradation | 8 | 13 |
| Glycolysis and Gluconeogenesis | 8 | 13 |
| Galactose metabolism | 8 | 12 |
| Histidine metabolism | 7 | 16 |
| Sialic acid metabolism | 7 | 15 |
| Fatty Acid Metabolism | 7 | 12 |
| Porphyrin metabolism | 7 | 12 |
| Glutamate metabolism | 7 | 10 |
| Pyruvate Metabolism | 7 | 10 |
| Glyoxylate and Dicarboxylate Metabolism | 7 | 8 |
| Vitamin E metabolism | 6 | 20 |
| Vitamin A (retinol) metabolism | 6 | 13 |
| Aminosugars metabolism | 6 | 13 |
| Glutathione Metabolism | 6 | 9 |
| Fructose and mannose metabolism | 6 | 8 |
| Beta-Alanine metabolism | 5 | 12 |
| Pentose phosphate pathway | 5 | 10 |
| Hexose phosphorylation | 5 | 7 |
| Vitamin B6 (pyridoxine) metabolism | 5 | 6 |
| Leukotriene metabolism | 4 | 13 |
| Vitamin B3 (nicotinate and nicotinamide) metabolism | 4 | 13 |
| Drug metabolism - other enzymes | 4 | 11 |
| Propanoate metabolism | 4 | 9 |
| Vitamin D3 (cholecalciferol) metabolism | 4 | 6 |
| Chondroitin sulfate degradation | 4 | 5 |
| Ubiquinone Biosynthesis | 4 | 5 |
| Heparan sulfate degradation | 4 | 5 |
| D4&E4-neuroprostanes formation | 4 | 4 |
| Squalene and cholesterol biosynthesis | 3 | 11 |
| Caffeine metabolism | 3 | 7 |
| Vitamin B1 (thiamin) metabolism | 3 | 6 |
| Selenoamino acid metabolism | 3 | 6 |
| Putative anti-Inflammatory metabolites formation from EPA | 3 | 5 |
| Carbon fixation | 3 | 4 |
| Vitamin H (biotin) metabolism | 3 | 4 |
| Starch and Sucrose Metabolism | 3 | 3 |
| Vitamin B9 (folate) metabolism | 2 | 6 |
| Biopterin metabolism | 2 | 6 |
| Glycosphingolipid biosynthesis - ganglioseries | 2 | 6 |
| Alkaloid biosynthesis II | 2 | 5 |
| Phytanic acid peroxisomal oxidation | 2 | 5 |
| Keratan sulfate degradation | 2 | 4 |
| Glycosphingolipid biosynthesis - globoseries | 2 | 4 |
| Pentose and Glucuronate Interconversions | 2 | 4 |
| Fatty acid oxidation, peroxisome | 2 | 4 |
| N-Glycan biosynthesis | 2 | 3 |
| 3-oxo-10R-octadecatrienoate beta-oxidation | 2 | 2 |
| Vitamin B12 (cyanocobalamin) metabolism | 2 | 2 |
| C5-Branched dibasic acid metabolism | 2 | 2 |
| Vitamin B5 - CoA biosynthesis from pantothenate | 1 | 4 |
| Omega-6 fatty acid metabolism | 1 | 4 |
| Prostaglandin formation from dihomo gama-linoleic acid | 1 | 4 |
| Omega-3 fatty acid metabolism | 1 | 4 |
| Lipoate metabolism | 1 | 4 |
| Di-unsaturated fatty acid beta-oxidation | 1 | 3 |
| Saturated fatty acids beta-oxidation | 1 | 3 |
| N-Glycan Degradation | 1 | 3 |
| Glycosphingolipid biosynthesis - lactoseries | 1 | 2 |
| Nitrogen metabolism | 1 | 2 |
| Keratan sulfate biosynthesis | 1 | 2 |
| O-Glycan biosynthesis | 1 | 2 |
| Polyunsaturated fatty acid biosynthesis | 1 | 2 |
| Glycosphingolipid biosynthesis - neolactoseries | 1 | 2 |
| Parathio degradation | 1 | 2 |
| Blood Group Biosynthesis | 1 | 2 |
| Hyaluronan Metabolism | 1 | 2 |
| Limonene and pinene degradation | 1 | 1 |
| Benzoate degradation via CoA ligation | 1 | 1 |
| Proteoglycan biosynthesis | 1 | 1 |
| Sphingolipid metabolism | 1 | 1 |
| Vitamin K metabolism | 1 | 1 |
| Dynorphin metabolism | 1 | 1 |
